# Early surgical management of traumatic dislocation of the tibialis posterior tendon: a case report and review of the literature

**DOI:** 10.1186/s13256-018-1872-z

**Published:** 2018-11-23

**Authors:** Yuzuru Sakakibara, Hideji Kura, Atsushi Teramoto, Toshihiko Yamashita

**Affiliations:** 1 Department of Orthopaedic Surgery, Sapporo Medical University, Sapporo, Japan; 2 Department of Orthopaedic Surgery, Hitsujigaoka Hospital, 004-0021, 3-1-10, Aoba Town, Atsubetu ward, Sapporo, Hokkaido Japan

**Keywords:** Das De procedure, Early surgery, Tibialis posterior tendon, Traumatic dislocation

## Abstract

**Background:**

Traumatic dislocation of the tibialis posterior tendon at the ankle is a rare injury. Some of these cases are misdiagnosed as ankle sprains and are not treated properly. In addition, because the conservative treatment is not as effective as the surgical treatment, it is essential that patients be diagnosed early so that proper surgical treatment can be performed. We report the early surgical management of traumatic dislocation of the tibialis posterior tendon.

**Case presentation:**

A 44-year-old Japanese man, who was a karate coach, was injured while acting as an umpire in a karate competition. On the same day of his injury, he came to our hospital. He complained of swelling and pain in the medial malleolus. Anterior dislocation of the tibialis posterior tendon was detected upon palpation. Magnetic resonance imaging showed the presence of anterior dislocation of the tibialis posterior tendon with retinaculum injury. Four days after the injury, we performed the Das De procedure as the surgical treatment. Three months after the surgery, the patient was able to participate in karate again.

**Conclusions:**

Dislocation of the tibialis posterior tendon is likely to be misdiagnosed, thus delaying the start of proper treatment. It is essential to diagnose the patient accurately by carefully assessing the physical symptoms manifested. Moreover, magnetic resonance imaging can also be used for better diagnosis, thereby leading to an early and proper surgical treatment.

## Background

Dislocation of the tibialis posterior tendon was first reported by Martius in 1874 [1]. This injury is rare, with fewer than 50 cases reported in the English-language literature. It often takes a long time before a patient is diagnosed and treated, because this injury is easily overlooked. Moreover, conservative treatment is not as effective as surgical treatment. We report a case of a 44-year-old man with traumatic dislocation of the tibialis posterior tendon that was diagnosed and managed early.

## Case presentation

Our patient was a 44-year-old Japanese man who worked as a karate coach. He had no other past medical history. He does not smoke and is a moderate alcohol drinker. He complained of pain and swelling on the medial aspect of his right ankle and had difficulty in ambulation. He was injured while acting as an umpire in a karate competition. While trying to avoid contact with a player, he stepped on the floor with his ankle dorsiflexed and with his knee flexed. He immediately felt pain and heard a snapping sound in his ankle. He was brought to the hospital on the same day because of difficulty in ambulation after the injury.

**Fig. 1:**
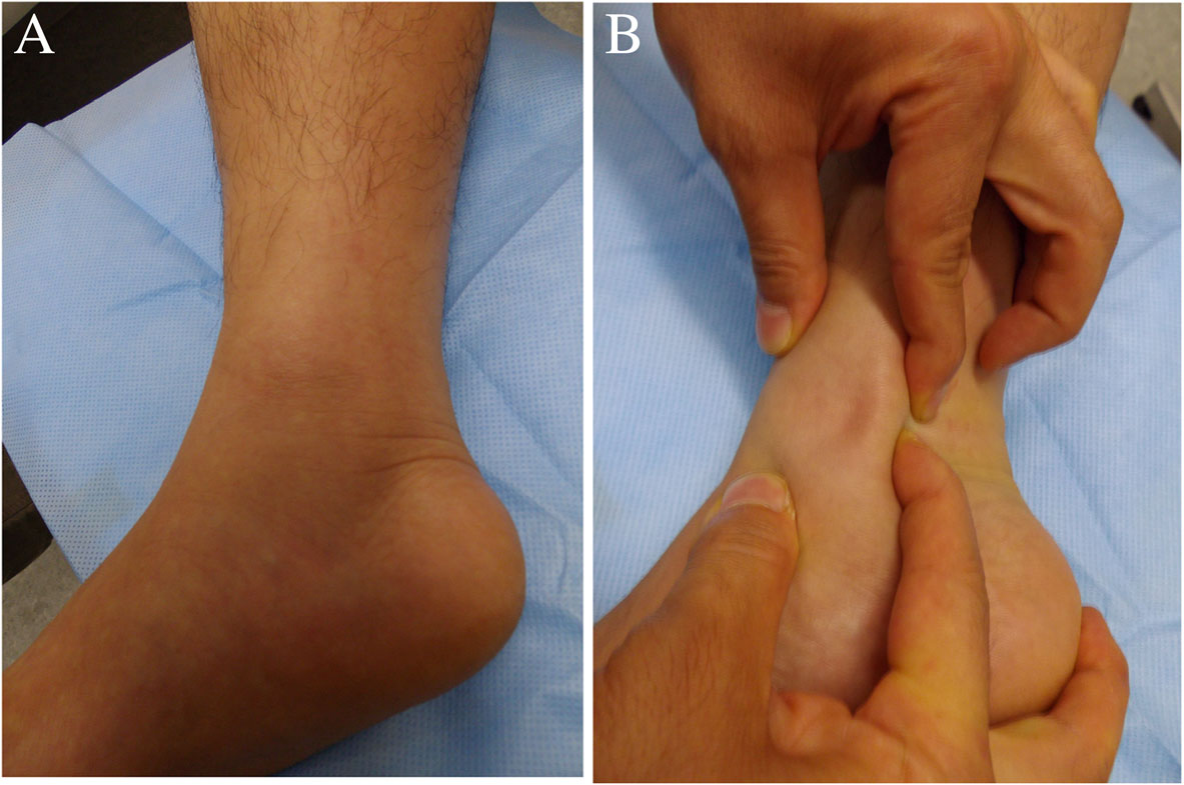
Appearance of the right ankle at the first visit. **a** Swelling on the medial side of the right ankle. **b** The tibialis posterior tendon is dislocated manually

No abnormality was observed in the laboratory data. Physical examination revealed that the medial side of his right ankle was swollen (Fig. 1a), and marked tenderness was present at the posterior of the medial malleolus. There was no ankle instability, as confirmed by the varus stress, valgus stress, and anterior drawer tests. The tibialis posterior tendon was dislocated and reduced manually with local anesthesia (1% xylocaine) around the medial malleolus (Fig. 1b). No neurological deficit was seen.

**Fig. 2:**
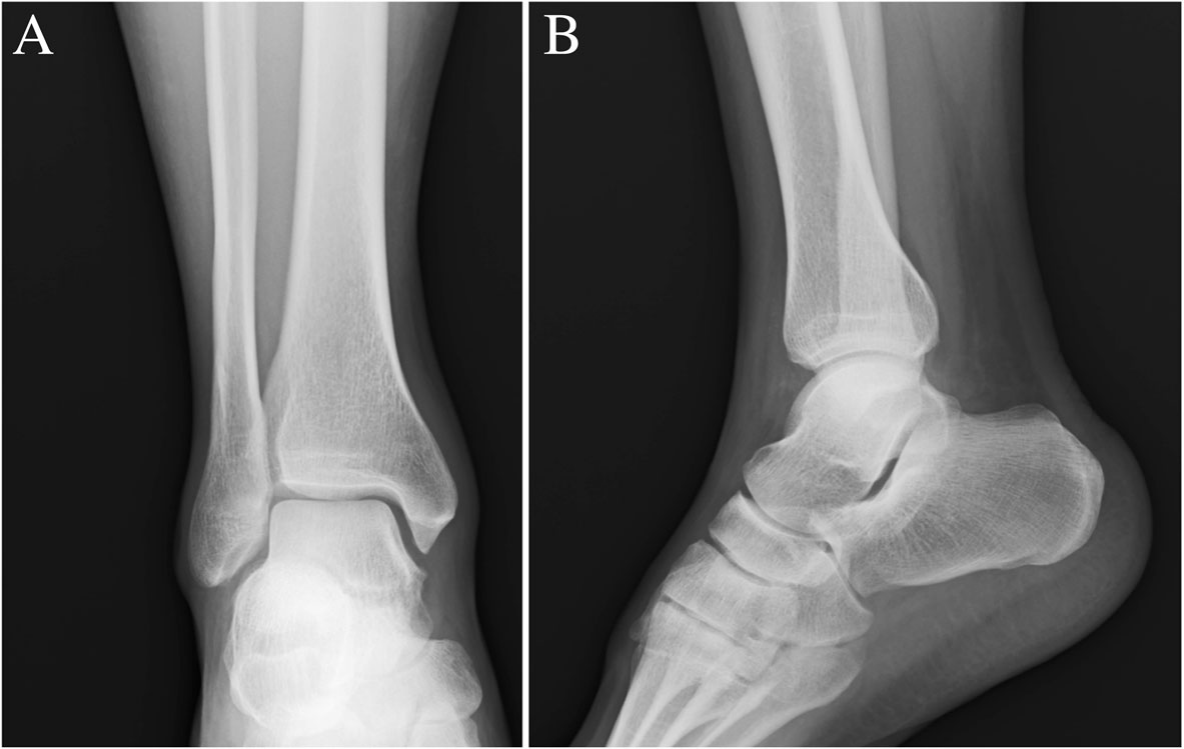
Standard radiographs of the ankle show a normal appearance. **a** Anteroposterior view. **b** Lateral view

**Fig. 3:**
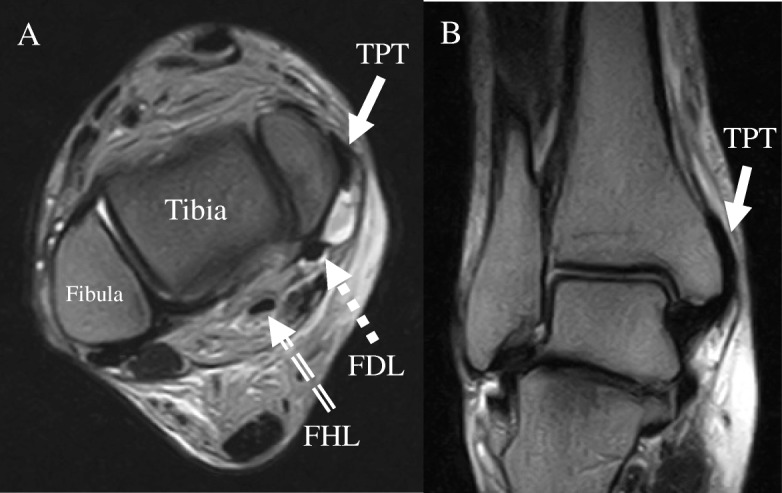
Magnetic resonance imaging demonstrates an anterior subluxated tibialis posterior tendon on the medial malleolus. Effusion is present at the retromalleolar groove. **a** T2-weighted MRI scan in the transverse plane. **b** T2-weighted MRI scan in the coronal plane. TPT (tibialis posterior tendon), FDL (flexor digitorum longus tendon), FHL (flexor hallucis longus tendon)

Standard radiographs showed a normal ankle appearance (Fig. 2). Magnetic resonance imaging (MRI) demonstrated an anterior subluxated tibialis posterior tendon that laid on the medial malleolus. Signal changes shown in the transverse plane of T2-weighted MRI scans revealed suspected fluid or bleeding at the retromalleolar groove (Fig. 3).

We diagnosed a dislocation of the tibialis posterior tendon based on the above-mentioned examination and performed surgical treatment at 4 days post-injury. Intraoperatively, the flexor retinaculum was detached from the medial malleolus, and a tendon sheath tear was noted. The tibialis posterior tendon was dislocated anteriorly from the medial malleolus groove. The tendon was torn longitudinally and sutured using 4-0 nylon. Drill holes were made in the medial malleolus using a 1.8-mm-diameter Kirschner wire following the Das De procedure [2]. The flexor retinaculum was attached to the bone at the medial malleolus, and the tendon sheath was repaired.

A below-the-knee cast kept the foot immobilized for 2 weeks. At 3 weeks, range-of-motion exercises for the ankle were started, and the patient was allowed to walk with an ankle-foot orthosis at 8 weeks. Subsequently, jogging was allowed at 12 weeks.

**Fig. 4:**
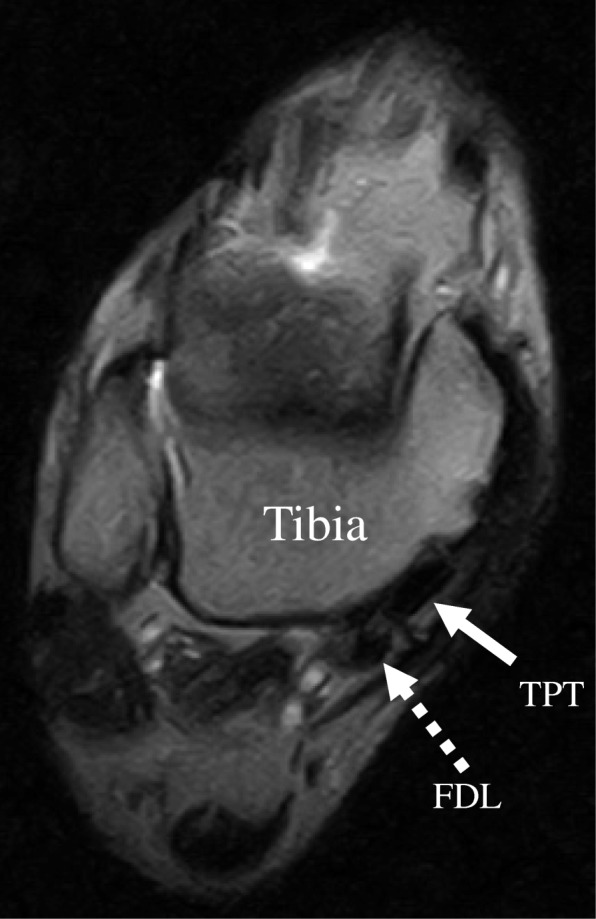
Latest 12-month postoperative magnetic resonance image shows the tibialis posterior tendon in a normal anatomical position. TPT (tibialis posterior tendon), FDL (flexor digitorum longus tendon)

At 1 year postoperatively, MRI showed that the tibialis posterior tendon was located at its normal anatomical position (Fig. 4), and the patient returned to work as a karate coach. The patient scored 100 on the Japanese Society for Surgery of the Foot ankle-hind foot scale (JSSF ankle-hind foot scale) (Table 1). No abnormal laboratory data or neurological deficit was present at 1 year postoperatively.

**Table 1:** Japanese Society for Surgery of the Foot ankle-hind foot scale

Parameter	Points
Pain (40 points)	
None	40
Mild	30
Moderate	20
Severe	0
Function (50 points)	
Activity limitations	
None	10
Limitations on recreational activities	7
Some limitations on daily and recreational activities	4
Severe limitations on daily and recreational activities	0
Maximum continuous walking distance	
600 m or more	5
400 m to less than 600 m	4
100 m to less than 400 m	3
Less than 100 m	0
Walking surface	
No difficulty on any surface	5
Some difficulty on uneven terrain, stairs, inclines	3
Severe difficulty or inability to walk on uneven terrain, stairs, inclines	0
Gait abnormality	
None or slight	8
Obvious (walking possible but gait) abnormality obvious)	4
Marked (walking difficult and gait) abnormality obvious)	0
Sagittal motion (flexion plus extension)	
Normal or mild restriction (30 degrees or more)	8
Moderate restriction (15–29 degrees)	4
Severe restriction (less than 15 degrees)	0
Hind foot motion (inversion plus eversion)	
Normal or mild restriction (75–100% normal)	6
Moderate restriction (25–74% normal)	3
Severe restriction (less than 25% normal)	0
Ankle-hind foot stability (anterior drawer, varus-valgus stress)	
Stable	8
Unstable	0
Alignment (10 points)	
Good, plantigrade foot, well aligned	10
Fair, plantigrade foot, mild to moderate degree of malalignment	5
Poor, nonplantigrade foot, severe malalignment	0

## Discussion and conclusions

Dislocation of the tibialis posterior tendon is uncommon. This injury is likely to be misdiagnosed; hence, it is important to make a diagnosis using careful physical and imaging examinations. We were able to make a diagnosis at the patient’s first visit as an outpatient. Moreover, we could perform early surgical treatment. An excellent result based on the JSSF ankle-hind foot scale was obtained 1 year after surgery because of the early diagnosis and early surgical treatment.

Traumatic dislocation of the tibialis posterior tendon is extremely rare. Approximately 50 cases have been reported in the English-language literature [3]. Upon a patient’s initial visit, a dislocated tibialis posterior tendon is often impalpable owing to marked swelling. Therefore, tibialis posterior tendon dislocation tends to be misdiagnosed as a deltoid ligament injury of the ankle [3–5]; thus, its correct diagnosis will still take several months [6, 7]. Traumatic tibialis posterior tendon dislocation is caused by (a) plantar flexion and inversion, (b) falling while the foot is in varus, (c) repeated forced inversion, or (d) twisting injuries and motor vehicle accidents [7, 8]. In our patient, the cause of the dislocation was forceful dorsiflexion and eversion.

For easy diagnosis, it is helpful for the tendon in front of the medial malleolus to be palpated. Local anesthesia (1% xylocaine) injected around the medial malleolus was useful during palpation of the dislocated tendon in our patient. Standard radiographs are necessary to rule out associated bone fractures. An internal rotation view is useful to detect a cortical avulsion at the insertion of the retinaculum [5, 6]. Computed tomography (CT) can also be helpful in detecting hypoplasia of the retromalleolar groove [4]. Ultrasonography is also important to understand the dynamic motion of the tendon [3, 4]. MRI is strongly recommended as the modality of choice for visualization of the dislocated tibialis posterior tendon and assessment of the flexor retinaculum [9].

**Fig. 5:**
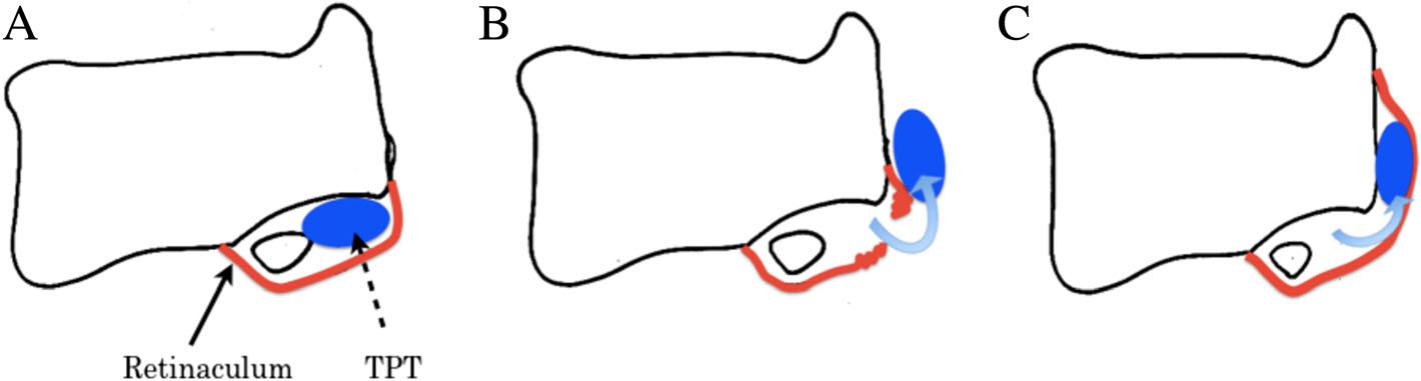
Schematic drawing of the tibialis posterior tendon (TPT) in an axial plane [3, 5]. **a** Normal anatomical features. **b** Subcutaneous dislocation of the TPT (Type 1). **c** Periosteal dislocation of the TPT (Type 2)

Two dislocation types were reported. Type 1 is subcutaneous dislocation due to anterior flexor retinaculum rupture. Type 2 is subperiosteal dislocation due to periosteal avulsion of the retinaculum at the insertion site with a periosteal flap to the medial malleolus. Type 2 is similar to the Bankart lesion in recurrent dislocation of the shoulder [3, 5]. Our patient was categorized as having a type 1 dislocation (Fig. 5).

Most authors have reported that conservative treatment is not as effective as surgical treatment [3–5, 7, 8]. Ouzounian *et al*. [10] described seven patients who had to undergo surgical treatment after a failed conservative treatment with casting, brace immobilization, and physical therapy. Surgical treatment should be considered even in an acute phase, because chronic dysfunction may cause potential complications, such as chronic pain, rupture of the tibialis posterior tendon, recurrent dislocation, and valgus deformity of the ankle [6].

The standard surgical treatment is tendon reduction and flexor retinaculum repair. If the retromalleolar groove is found to be hypoplastic, an additional procedure (deepening of the sulcus or bone grafting) is considered. Soler *et al.* [11] reported a wide variability in the size of the retromalleolar groove of the normal cadaveric ankle, with measurements ranging from 6 to 15 mm in width and 1.5 to 4 mm in depth. Preoperative CT is helpful to decide the appropriate surgical plan for the retromalleolar groove. The retromalleolar groove in our patient was 9 mm in width and 2 mm in depth, but hypoplasia of the retromalleolar groove was not present. Sufficient stability of the tibialis posterior tendon was achieved through flexor retinaculum repair only.

In athletes, the delay of the diagnosis and surgical treatment postpones recovery of participation in sports activity. Thus, a careful history and physical examination is important for the diagnosis. When dislocation of the tibialis posterior tendon is suspected at the first visit, it is necessary to order an additional imaging examination, such as MRI or ultrasonography, for the early diagnosis. Given the early diagnosis and surgical treatment of our patient, he may return early to sports activity and obtain an excellent functional score.

## Abbreviations

CT: Computed tomography
JSSF ankle-hind foot scale: Japanese Society for Surgery of the Foot ankle-hind foot scale
MRI: Magnetic resonance imaging
